# A Machine Learning–Based Approach to Discrimination of Tauopathies Using [ ^18^F]PM‐PBB3 PET Images

**DOI:** 10.1002/mds.29173

**Published:** 2022-08-28

**Authors:** Hironobu Endo, Kenji Tagai, Maiko Ono, Yoko Ikoma, Asaka Oyama, Kiwamu Matsuoka, Naomi Kokubo, Kosei Hirata, Yasunori Sano, Masaki Oya, Hideki Matsumoto, Shin Kurose, Chie Seki, Hiroshi Shimizu, Akiyoshi Kakita, Keisuke Takahata, Hitoshi Shinotoh, Hitoshi Shimada, Takahiko Tokuda, Kazunori Kawamura, Ming‐Rong Zhang, Kenichi Oishi, Susumu Mori, Yuhei Takado, Makoto Higuchi

**Affiliations:** ^1^ Institute for Quantum Medical Science, Quantum Life and Medical Science Directorate National Institutes for Quantum Science and Technology Chiba Japan; ^2^ Department of Oral and Maxillofacial Radiology Tokyo Dental College Tokyo Japan; ^3^ Department of Pathology, Brain Research Institute Niigata University Niigata Japan; ^4^ Neurology Clinic Chiba Chiba Japan; ^5^ Department of Functional Neurology & Neurosurgery, Center for Integrated Human Brain Science, Brain Research Institute Niigata University Niigata Japan; ^6^ The Russell H. Morgan Department of Radiology and Radiological Science Johns Hopkins University School of Medicine Baltimore Maryland USA

**Keywords:** Alzheimer's disease, machine learning, progressive supranuclear palsy, tauopathy, tau PET

## Abstract

**Background:**

We recently developed a positron emission tomography (PET) probe, [^18^F]PM‐PBB3, to detect tau lesions in diverse tauopathies, including mixed three‐repeat and four‐repeat (3R + 4R) tau fibrils in Alzheimer's disease (AD) and 4R tau aggregates in progressive supranuclear palsy (PSP). For wider availability of this technology for clinical settings, bias‐free quantitative evaluation of tau images without a priori disease information is needed.

**Objective:**

We aimed to establish tau PET pathology indices to characterize PSP and AD using a machine learning approach and test their validity and tracer capabilities.

**Methods:**

Data were obtained from 50 healthy control subjects, 46 patients with PSP Richardson syndrome, and 37 patients on the AD continuum. Tau PET data from 114 regions of interest were subjected to Elastic Net cross‐validation linear classification analysis with a one‐versus‐the‐rest multiclass strategy to obtain a linear function that discriminates diseases by maximizing the area under the receiver operating characteristic curve. We defined PSP‐ and AD‐tau scores for each participant as values of the functions optimized for differentiating PSP (4R) and AD (3R + 4R), respectively, from others.

**Results:**

The discriminatory ability of PSP‐ and AD‐tau scores assessed as the area under the receiver operating characteristic curve was 0.98 and 1.00, respectively. PSP‐tau scores correlated with the PSP rating scale in patients with PSP, and AD‐tau scores correlated with Mini‐Mental State Examination scores in healthy control–AD continuum patients. The globus pallidus and amygdala were highlighted as regions with high weight coefficients for determining PSP‐ and AD‐tau scores, respectively.

**Conclusions:**

These findings highlight our technology's unbiased capability to identify topologies of 3R + 4R versus 4R tau deposits. © 2022 The Authors. *Movement Disorders* published by Wiley Periodicals LLC on behalf of International Parkinson and Movement Disorder Society

## Graphical abstract

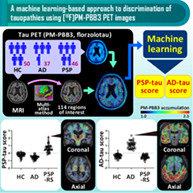

Diverse neurodegenerative disorders are neuropathologically characterized by depositions of tau protein fibrils and are collectively referred to as tauopathies.^1^, ^2^, ^3^ Tauopathies are classified according to the composition of the tau isoform forming the aggregates.^4^ Alzheimer's disease (AD) mainly presents with memory impairments and accumulations of mixed three‐repeat and four‐repeat (3R + 4R) tau isoforms, while progressive supranuclear palsy (PSP) is noted with motor symptoms and accumulations of 4R tau isoforms.^5^, ^6^, ^7^ During the early course of AD, 3R + 4R tau lesions are observed in the entorhinal cortex and hippocampus of the medial temporal lobe; as the disease progresses, these lesions spread to the neocortex.^8^ It is assumed that the spread of tau lesions is aggravated by the coexistence of amyloid‐β (Aβ) pathology.^9^ In PSP, it is assumed that the 4R tau protein aggregation of pallido‐luyso‐nigral region in the early stage of the disease spreads to the striatum, cerebellar dentate nucleus, and frontoparietal lobe.^10^ Because the accumulation of tau aggregates is associated with neurodegeneration,^11^ tau lesions are an important target for the development of therapeutic agents in tauopathies.

Although each tauopathy has several unique clinical features, diagnosis and severity assessment based on pathogenesis remain difficult, especially in the early stages of the disease. Quantification of tau lesions in the brain is necessary to evaluate the efficacy of treatment of tauopathy. Positron emission tomography (PET) is a technique that can visualize tau lesions in the in vivo brain using various probes. We developed the [^18^]PM‐PBB3 ([^18^F]APN‐1607; [^18^F]florzolotau) probe to capture 3R + 4R tau lesions in AD and 4R tau lesions in PSP.^12^ Although various probes for tauopathies have been previously developed, they were not able to detect non‐AD tauopathies at the individual level.^13^, ^14^, ^15^, ^16^, ^17^, ^18^, ^19^, ^20^, ^21^, ^22^ [^18^F]PM‐PBB3 provides better contrast for group comparison than any currently available probe. Specifically, the ratio of the PSP group to healthy controls (HCs) for target/reference [^18^F]PM‐PBB3 binding^12^ exceeded 1.5, whereas the ratio for other probes was less than 1.2.^22^, ^23^, ^24^

The presence of 3R + 4R and 4R tau lesions may be distinguished by examining PET with [^18^F]PI‐2620, a second‐generation analog of a widely used tau probe, [^18^F]flortaucipir, based on a nonclinical study. However, clinical PET assays have reported that the performance of [^18^F]PI‐2620 for the discrimination between PSP cases and controls may not markedly exceed that of [^18^F]flortaucipir.^22^, ^25^, ^26^

To use [^18^F]PM‐PBB3 as a disease index or for therapeutic monitoring, it is first necessary to characterize the disease‐specific tau accumulation pattern. Two technologies are essential for such characterization: image quantification and pattern recognition. For image quantification, the current gold standard is to manually define the regions of interest (ROIs). However, this approach requires a great deal of effort to analyze large datasets in clinical trials. The commonly used qualitative evaluation method also has issues related to arbitrariness and reproducibility. Therefore, automatic quantitative methods using computer programs are increasingly used, and automatic whole‐brain ROI methods have been developed.^27^, ^28^ This method automatically identifies brain regions and performs parcel‐based analysis (PBA) for each region. This analysis approach is suitable for detecting subtle changes in defined ROIs throughout the brain on PET images, which can be understood based on the known neuroanatomical functions of the segmented regions. A multi‐atlas method has been developed that automatically segments the brain for more accurate PBA.^29^, ^30^ It uses multiple brain atlases as supervisory data and identifies the brain structure of each subject's image by machine learning. The application of this method to PET images has the potential to reduce misregistration during brain deformation and to achieve the accuracy required in clinical trials.

Once an image has been quantified, the next step is to perform pattern recognition to extract information relevant to diagnosis and treatment. When a data‐driven approach is used to identify a novel index for diagnosis or treatment, pattern recognition often requires a machine learning framework with a validation step. The Elastic Net is one of the machine learning methods.^31^ Not only does this method select effective brain regions from a large number of features (in this case, tau PET accumulation in segmented brain regions) and weight them, it also reduces dimensionality by setting the coefficients to zero for brain regions with low contributions. Furthermore, it enables multicollinearity to be considered in the analysis. This is important because when several features with tau PET accumulation in segmented brain regions are highly correlated, the regression between them can be unstable. For example, when PBA is performed for a brain region such as the basal ganglia, there are data for the left and right hemisphere regions, which have high multicollinearity. The Elastic Net analysis enables the creation of a robust classification model based on a small number of tau PET datasets and achieves high interpretability and explanatory ability.

The goal of this study was to test the validity of the machine learning approach and the ability of the [^18^F]PM‐PBB3 tracer to identify and characterize tau pathology in two distinct neurodegenerative disorders: namely, AD (3R + 4R tauopathy) and PSP (4R tauopathy). We aimed to create a biomarker that can reflect the difference in the distribution of tauopathies between PSP and AD that can be used to evaluate the efficacy of existing anti‐tau lesion treatment seeds. For this purpose, we checked whether the developed PET pathology index for AD and PSP can detect the known distribution of pathology.

## Subjects and Methods

### Participants

Between May 2017 and September 2021, 46 patients with probable PSP Richardson syndrome (PSP‐RS), 11 with PSP‐non‐RS (including 7 with probable PSP‐parkinsonism [PSP‐P], 1 with possible PSP‐pure akinesia with gait freezing, 2 suggestive of PSP‐P, and 1 suggestive of PSP‐frontal lobe cognitive or behavioral presentations), and 37 with AD continuums (including 12 with Aβ‐positive mild cognitive impairment [MCI]) were recruited from affiliated hospitals. Aβ‐positive was defined as per [^11^C] Pittsburgh Compound B ([^11^C]PiB) PET visual inspection performed by at least three experienced PET specialists.^32^ Diagnosis of PSP was based on the International Parkinson and Movement Disorder Society clinical diagnostic criteria for PSP,^33^ whereas that of AD continuum (hereafter AD) was based on Petersen's criteria^34^ and the National Institute of Neurological and Communicative Diseases and Stroke/Alzheimer's Disease and Related Disorders Association criteria.^35^

In addition, 50 HCs aged older than 40 years, without histories of neurological disorders, Aβ‐negative as per [^11^C]PiB PET by the visual inspection described earlier, and with a Mini‐Mental State Examination (MMSE) score of ≥28 or Montreal Cognitive Assessment score of ≥26 and Geriatric Depression Scale score of ≤5 or no history of depression were recruited from the volunteer association of our institute. The neuropsychological tests performed are described in detail in the eMethods section in Appendix S1.

Written informed consent was obtained from all participants and/or spouses or other close family members if the participants were cognitively impaired. This study was approved by the Radiation Drug Safety Committee and National Institutes for Quantum Science and Technology Certified Review Board of Japan. The study was registered with UMIN Clinical Trials Registry (numbers 000026385, 000029608, 000026490, 000030248, and 000043458).

### Magnetic Resonance Imaging Studies

Participants underwent magnetic resonance (MR) scanning on 3‐T MAGNETOM Verio (SIEMENS). T1‐weighted gradient‐echo sequence images were taken for coregistration and segmentation of PET images (sagittal orientation; 1‐mm‐thick sections; echo time [TE], 1.95 ms; repetition time [TR], 2300 ms; flip angle, 9.0; inversion time [TI], 900 ms; field of view [FOV], 250 mm; matrix size, 512 × 512 × 176).

### PET Studies

The radiosynthesis of [^18^F]PM‐PBB3 and [^11^C]PiB is described in the Supporting Information (eMethods in Appendix S1).^36^, ^37^ PET scans were performed with a Biograph mCT flow system (Siemens Healthcare; matrix size 200 × 200 × 109; voxel size [mm] 2 × 2 × 2 for [^18^F]PM‐PBB3 and [^11^C]PiB ligands) and a discovery MI (GE Medical Systems; matrix size 128 × 128 × 89; voxel size [mm] 2 × 2 × 2.8 for [^11^C]PiB ligand). PET images were reconstructed using a filtered back‐projection algorithm with a Hanning filter (6.0 mm full width at half maximum). Detailed PET scanning protocols were described previously.^12^, ^38^ Each participant received 90‐ to 110‐minute PET scans (frames: 4 × 5 or 2 × 10 minutes) after intravenous injection of [^18^F]PM‐PBB3 (186 ± 8.4 MBq) and 50‐ to 70‐minute PET scans (frames: 4 × 5) after intravenous injection of [^11^C]PiB (526 ± 72.0 MBq) within 3 months.

### Histological Examination

We performed triple staining with PM‐PBB3, Gallyas‐Braak, and AT8 antibodies in HC, PSP‐RS, and AD brain sections from different studies to confirm tau accumulation in regions that were important for discrimination as determined by machine learning. Details of the pathological section preparation and participants are described in the eMethods section in Appendix S1.

### Data Preprocessing and the Definition of ROIs on PET Images

The details of data preprocessing are described in the eMethods in Appendix S1. The whole brain was automatically parcellated into 287 regions using the M‐Vision brain (M Corporation, Tokyo, Japan), a standalone version of the MR brain image analysis system: BrainGPS. The BrainGPS was developed by K.O., S.M., and colleagues at the Department of Radiology, Johns Hopkins University, and licensed to AnatomyWorks, LLC. It is a magnetic resonance imaging (MRI) analysis cloud platform developed based on a technique (MRIcloud: https://mricloud.org/) that warps multiple atlases into linearly arranged images using Large Deformation Diffeomorphic Metric Mapping,^29^ followed by a multi‐atlas fusion algorithm.^30^ The M‐Vision brain can be used efficiently and safely within a closed laboratory, and performance is comparable with MRIcloud (BrainGPS). We followed the structural definition of the Johns Hopkins University brain atlas^39^ and identified brain structures at five hierarchical levels to obtain comprehensive information on brain morphology. In this study, we created a parcellation map using the expectation–maximization algorithm, which is a machine learning method, using the “Elderly (>50y) V.10A 29 atlases” implemented in M‐Vision brain. We used 114 ROIs of brain parenchyma among 144 ROIs in the fourth hierarchical level for PET data measurements.

### Machine Learning Analysis for Recognition of Disease‐Specific Patterns

The Elastic Net model was applied to PET data to generate a model to provide the PET pathology indices that can identify two types of tauopathy: PSP‐RS and AD. The standardized uptake value ratios (SUVRs) of 114 target ROIs and the gray matter reference region were quantified without partial volume correction. The gray matter reference region was the area identified by the histogram of tau accumulation as the region of low tau accumulation.^38^ All SUVRs were corrected for age and sex (linear regression) and standardized (mean = 0; standard deviation = 1) using HC, AD, and PSP‐RS data.

The data were randomly split 10 times into two sets: one for training (75%) and the other for diagnostic performance evaluation (25% of test data). Data were split using the “stratified shuffle split” function in Python's scikit‐learn library with a constant sex ratio (Fig. S1A).^40^ Elastic Net parameter optimization was performed as “test 1,” the linear combination with SUVR values using the averaged weighting coefficients was performed on test data to evaluate the diagnostic performance as “test 2” (Fig. S1D), and the linear combination with SUVR values using the averaged weighting coefficients was performed on all data before splitting for receiver operating characteristic (ROC) analysis as “test 3” (Fig. S1E). Details of Elastic Net (including parameters L1_ratio and *α*) are provided in the Supporting Information (eMethods in Appendix S1).

For test 1, the optimized weight coefficients of the linear function for discrimination were obtained through machine learning (Elastic Net: sklearn.linear_model.enet_path; fivefold cross‐validation) so that the area under the ROC curve (AUC) of the PSP‐RS group versus the HC + AD group by SUVR of each area was maximized in the 10 training datasets and the discrimination ability was highest.^31^, ^40^ The coefficients of the ROIs important for discrimination were determined using the mean L1_ratio and the individual optimal α of the 10 randomly split datasets. The coefficients of each of these ROIs were averaged for the 10 datasets and adapted to the test data. For test 2, the sum of the obtained weight coefficients multiplied by the SUVR of each ROI was defined as the PSP‐tau score, which was calculated by applying the learned weight coefficients to the SUVR of the test data to evaluate the diagnostic performance. The same process was performed for the AD group versus the HC + PSP‐RS group to obtain the AD‐tau score. For the HC group, we also calculated the PSP‐tau score and AD‐tau score by linear combinations using each coefficient calculated earlier and the individual SUVR. The correlation between the obtained score and the severity of the disease was also analyzed.

### Statistical Analysis

We used analysis of variance for comparison of age and the chi‐squared test with Bonferroni correction for sex between the HC, AD, PSP‐RS, and non‐PSP‐RS groups. To examine correlations between tau scores and clinical scores, we used Spearman's rho test. Pearson's test was used to determine the correlation between tau scores and disease duration. The remaining demographic characteristics were analyzed using the Kruskal–Wallis test with post hoc Dunn's test. To assess diagnostic performance, including the accuracy, sensitivity, and specificity of differentiation between PSP‐RS and (HC + AD) or AD and (HC + PSP‐RS), ROC curve analysis of the PSP‐tau score and AD‐tau score was performed. We applied the Youden index to determine the optimal cutoff tau score value for separating every two diagnostic groups. The range of statistical significance was defined as *P* < 0.05. We used Prism software (version 8.4.3; GraphPad Software, San Diego, CA, USA) for all statistical analyses. All values are reported as mean ± standard deviation.

### Data Sharing

Requests for data that support the findings of this study should be directed to corresponding authors, Hironobu Endo (endo.hironobu@qst.go.jp) and Yuhei Takado (takado.yuhei@qst.go.jp), and will be available on reasonable request.

## Results

Demographics data are presented in Table 1. In test 1, the Elastic Net parameters of the PSP‐tau score were L1_ratio of 0.457 ± 0.389 and *α* of 0.164 ± 0.098 (see eMethods in Appendix S1 for details of these parameters; Fig. S1A). The mean AUC of the 10 training datasets was 0.963 ± 0.008. In contrast, the Elastic Net parameters of the AD‐tau score were L1_ratio of 0.543 ± 0.424 and *α* of 0.129 ± 0.148, and the AUC was 0.998 ± 0.002.

**TABLE 1:** Demographics of individuals included in this study

Demographics	HC	PSP‐RS	AD continuum	PSP‐non‐RS	*P* values
n (race: all Asian)	50	46	37	11	
Age (y)	64.0 ± 11.5	71.4 ± 7.5**	69.1 ± 11.0	71.7 ± 6.2	0.005
Male sex, n (%)	29 (58)	28 (61)	15 (41)	6 (55)	0.27
Amyloid‐β positive	0	0	37	0	
Disease duration (y)		3.4 ± 2.5	2.7 ± 2.2	3.3 ± 2.0	0.2
UPDRS Part III score	1.4 ± 2.4 (*n* = 40)	34.2 ± 14.5****^,####^ (*n* = 43)	2.2 ± 4.4 (*n* = 29)	21.5 ± 9.0***^,##^	<0.0001
PSP rating scale	1.8 ± 2.0 (*n* = 20)	40.0 ± 17.4****^,##^	4.7 ± 1.0 (*n* = 6)	20.6 ± 9.2*	<0.0001
MMSE	29.3 ± 0.9	23.8 ± 6.5**** (*n* = 43)	21.6 ± 3.5**** (*n* = 36)	26.4 ± 3.2*^,#^	<0.0001
MoCA	27.4 ± 2.1 (*n* = 15)	22.6 ± 3.9 (*n* = 5)	15.3 ± 4.7**** (*n* = 7)	26^a^ (*n* = 1)	<0.0001
FAB	16.4 ± 1.3 (*n* = 48)	11.6 ± 3.8**** (*n* = 40)	12.5 ± 3.4**** (*n* = 32)	12.6 ± 3.2***	<0.0001
GDS	1.6 ± 1.2 (*n* = 32)	6.4 ± 4.1**** (*n* = 39)	4.3 ± 2.8*** (*n* = 34)	5.5 ± 2.0****	<0.0001
Apathy	9.5 ± 5.4 (*n* = 38)	17.9 ± 8.9**** (*n* = 38)	14.7 ± 7.4** (*n* = 35)	17.6 ± 6.7*	<0.0001
CDR	NE	0.7 ± 0.7^#^ (*n* = 14)	0.9 ± 0.5 (*n* = 36)	0.5 ± 0.3^#^ (*n* = 9)	0.004
CDR‐SoB	NE	4.7 ± 4.6 (*n* = 14)	4.1 ± 2.8 (*n* = 36)	1.8 ± 1.4^#^ (*n* = 9)	0.04

****P < 0.0001, ***P <0.001, **P <0.01, *P < 0.05 versus HC post hoc Dunn's test; ^####^ *P* < 0.0001, ^###^ *P* < 0.001, ^##^ *P* < 0.01, ^#^ *P* < 0.05 versus AD continuum post hoc Dunn's test.

^a^ Statistics not examined.

HC, healthy control; PSP‐RS, progressive supranuclear palsy–Richardson's syndrome; AD, Alzheimer's disease; PSP‐non‐RS, progressive supranuclear palsy without Richardson's syndrome; UPDRS III, Unified Parkinson's Disease Rating Scale Part III; MMSE, Mini‐Mental State Examination; MoCA, Montreal Cognitive Assessment; FAB, Frontal Assessment Battery; GDS, Geriatric Depression Scale; CDR, Clinical Dementia Rating scale; NE, not examined; CDR‐SoB, Clinical Dementia Rating scale Sum of Boxes.

**FIG 1:**
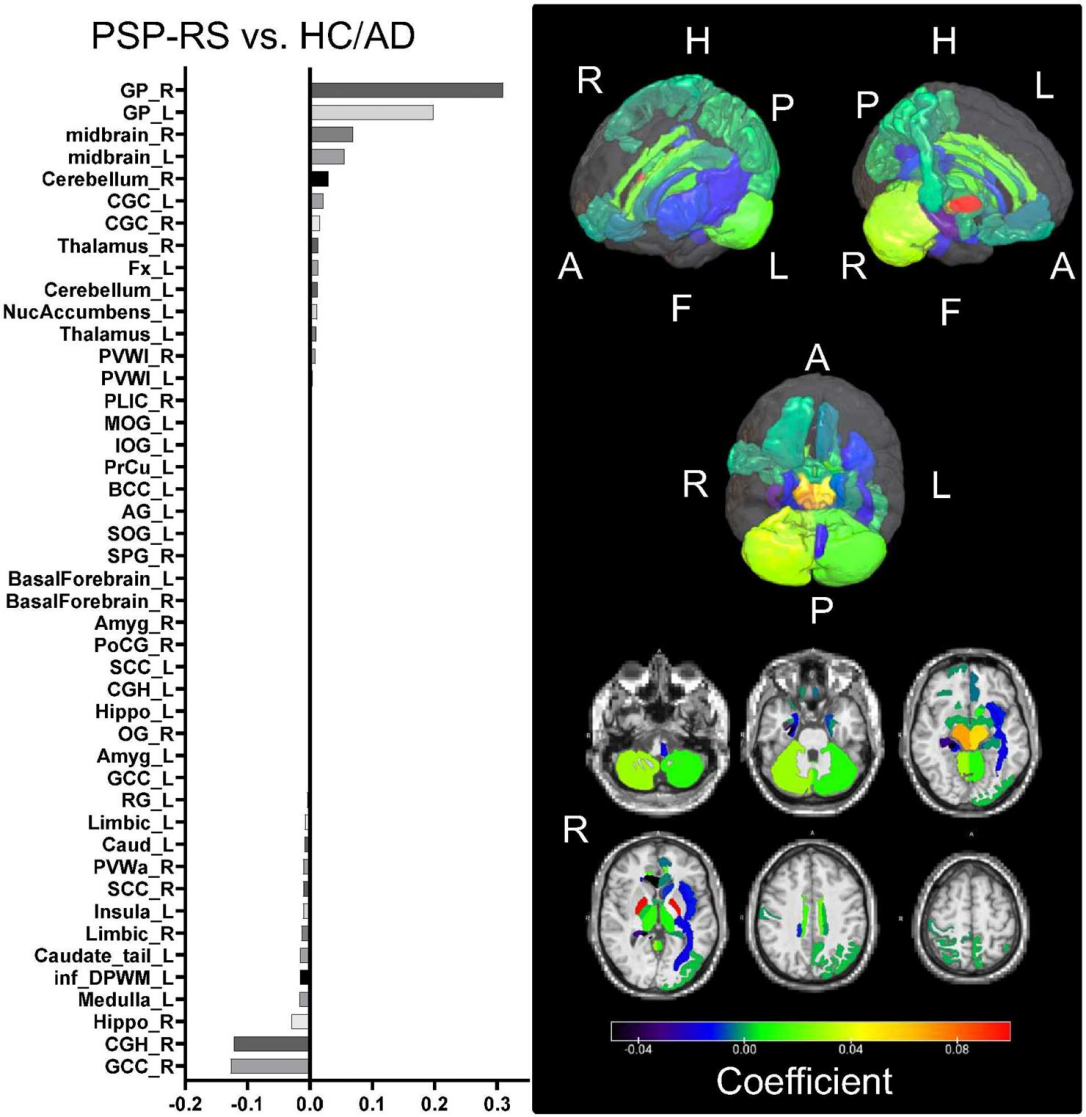
Weighting coefficients for the ROIs that contributed most to the discrimination between the PSP‐RS and AD + HC groups. The bar chart on the left shows the important ROIs from the top in the order of positive to negative weighting coefficients. In the right panel, the upper panel shows the weighting coefficients assigned to the ROIs in the three‐dimensional brain, and the lower panel shows the axial cross‐section of the ROIs, with the GP and midbrain having higher weighting coefficients, from red to yellow. A, Anterior; AD, Alzheimer's disease; AG, angular gyrus; Amyg, amygdala; BCC, body of corpus callosum; Caud, caudate nucleus; CGC, cingulum (cingulate gyrus); CGH, cingulum (hippocampus gyrus); F, foot; Fx, fornix (column and body of fornix); GCC, genu of corpus callosum; GP, globus pallidus; H, head; HC, healthy control; Hippo, hippocampus; inf_DPWM, inferior deep white matter; IOG, inferior occipital gyrus; L, left; MOG, middle occipital gyrus; NucAccumbens, nucleus accumbens; OG, orbital gyrus; P, posterior; PLIC, posterior limb of internal capsule; PoCG, postcentral gyrus; PrCu, pre‐cuneus; PSP‐RS, progressive supranuclear palsy–Richardson syndrome; PVWa, periventricular white matter anterior; PVWl, periventricular white matter lateral; R, right; RG, gyrus rectus; ROI, region of interest; SCC, splenium of corpus callosum; SOG, superior occipital gyrus; SPG, superior parietal gyrus. See also Table S1 for the abbreviations in the left graph. [Color figure can be viewed at wileyonlinelibrary.com]

**FIG 2:**
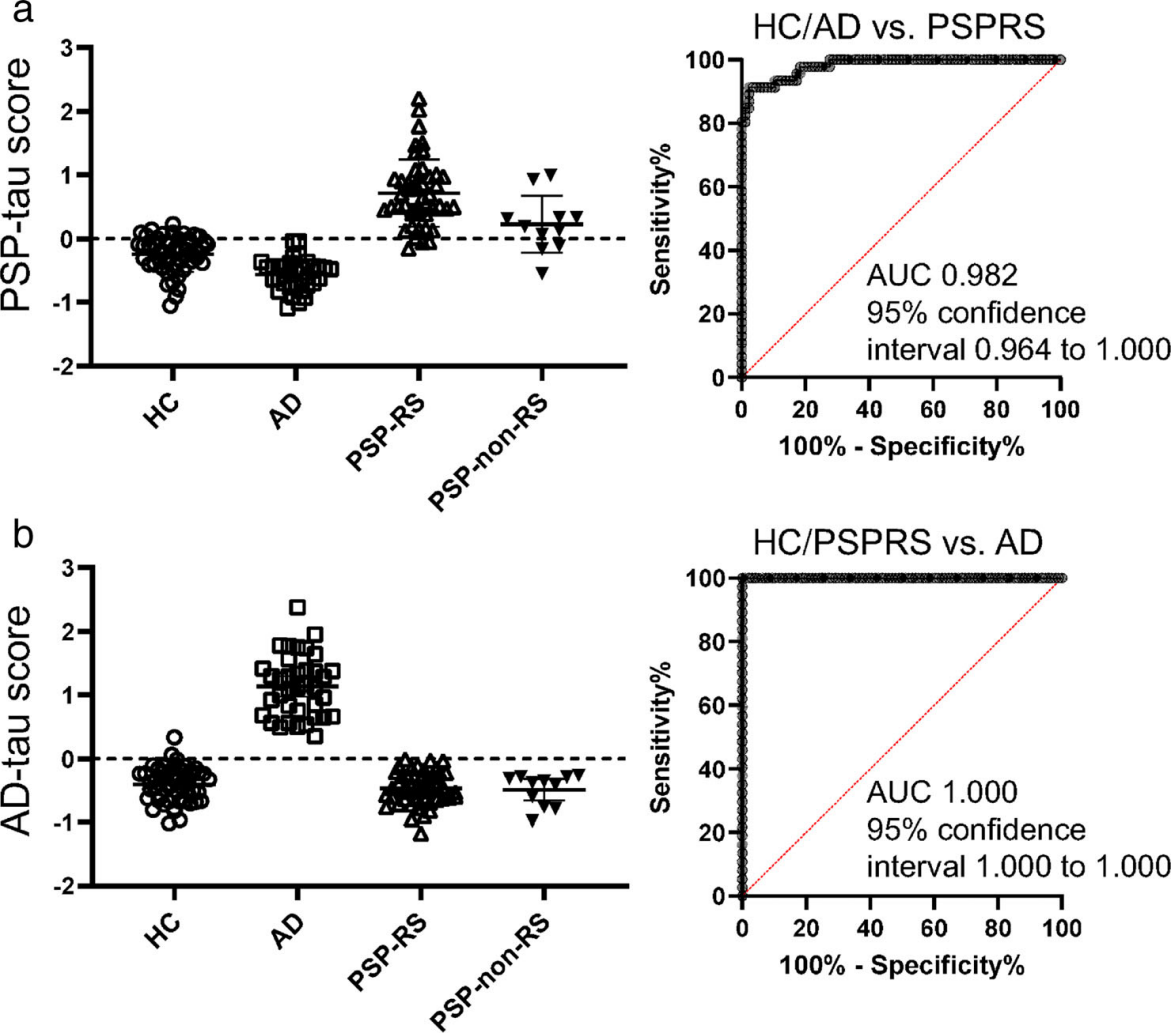
Plots of PSP‐tau score and AD‐tau score in each group and AUC for the ROC analysis. (**A**) Figure plotted with PSP‐tau score on the vertical axis and HC (circle), AD (square), PSP‐RS (triangle), and PSP‐non‐RS (filled reverse triangle) on the horizontal axis. The dotted line indicates the zero line. The rightmost group shows the weighting coefficients trained on HC+ AD and PSP‐RS, which were also applied to PSP‐non‐RS. (**B**) The vertical axis is AD‐tau score; the horizontal axis shows HC, AD, PSP‐RS, and non‐PSP‐RS as in (**A**), respectively. The dotted line indicates the zero line. In the rightmost group, the weighting coefficients trained for HC + PSP‐RS and AD were also applied to PSP‐non‐RS. AD, Alzheimer's disease; AUC, area under the curve; HC, healthy control; PSP, progressive supranuclear palsy; PSP‐non‐RS, progressive supranuclear palsy without Richardson's syndrome; PSP‐RS, progressive supranuclear palsy–Richardson's syndrome; ROC, receiver operating characteristic. [Color figure can be viewed at wileyonlinelibrary.com]

The optimized averaging coefficients important for discriminating the PSP‐RS group versus the AD + HC group (= PSP‐tau score coefficient) were positive in the globus pallidus (GP) and midbrain (the higher the tau accumulation in ROIs with positive coefficients, the higher the score) and negative in the cingulum segment adjoining the hippocampus (including the parahippocampal gyrus and entorhinal cortex) and genu of the corpus callosum (Fig. 1); in contrast, those important for discriminating the AD group versus the PSP‐RS + HC group (= AD‐tau score coefficient) were positive in the medial temporal lobe, including the amygdala and cingulum, and negative in the cerebellum (Fig. S2). In test 2 (Fig. S1D), the diagnostic performance of the PSP‐tau score and AD‐tau score showed 95.0% ± 0.02% accuracy, 87.6% ± 0.05% sensitivity, and 98.6% ± 0.02% specificity with a cutoff > 0.1986 for PSP‐RS discrimination, and 98.6% ± 0.02% accuracy, 100% sensitivity, and 100% specificity with a cutoff > 0.3431 for AD discrimination. In test 3 (Fig. S1E), the AUC for the PSP‐RS group versus the AD + HC group was 0.982, and that for the AD group versus the PSP‐RS + HC group was 1.000 (Fig. 2). Representative figures of cases with high and low scores are shown in the Supporting Information (Fig. S4).

**FIG 3:**
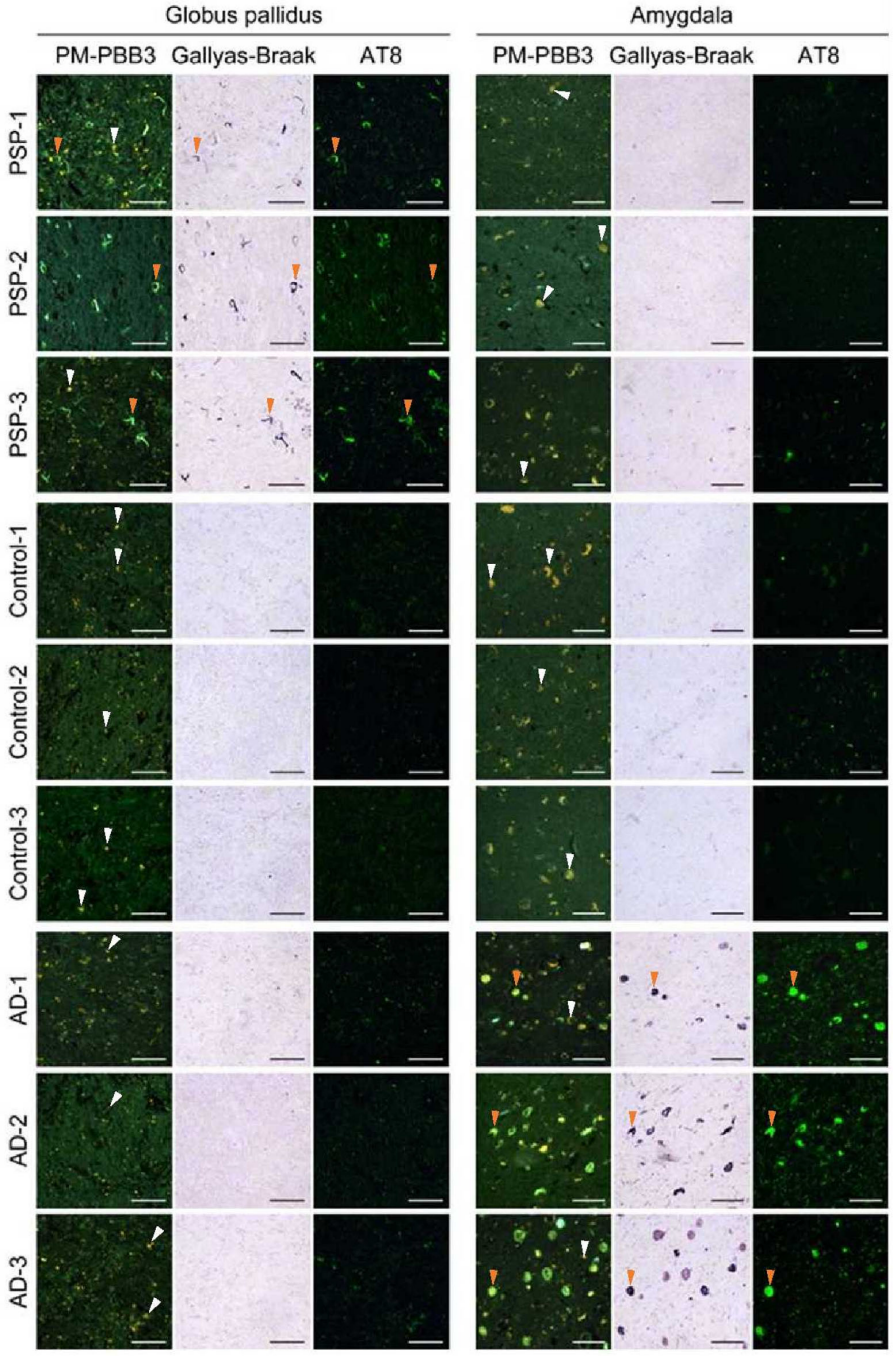
Triple pathological staining of autopsy cases of different studies in PSP‐RS and AD. The three columns on the left show the pathological sections of the GP, which was the most important region for the discrimination of PSP‐RS, and that were stained with PM‐PBB3, Gallyas‐Braak, and AT8 antibodies. The top three rows show the PSP‐RS, HC, and AD groups, with PSP‐RS presented from the lowest to highest PSP rating scale. The three right columns show the pathological sections of the amygdala, which was the most important region for the discrimination of AD, triple‐stained in the same way. The images show that tau was well captured by the three stains in the GP of PSP‐RS cases and the amygdala in AD cases. PM‐PBB3 staining shows green for fluorescence and yellow for autofluorescence. The orange arrowhead indicates a typical tau lesion consistent with triple staining, whereas the white arrowhead indicates typical autofluorescence. AD, Alzheimer's disease; GP, globus pallidus; HC, healthy control; PSP‐RS, progressive supranuclear palsy–Richardson's syndrome. [Color figure can be viewed at wileyonlinelibrary.com]

Triple pathological staining of autopsy cases prepared separately from this study showed that PM‐PBB3–positive tau lesions were present in large amounts in the GP sections of patients with PSP but rarely in the GP sections of patients with AD and HCs, and in large amounts in the amygdala sections of patients with AD but rarely in the amygdala sections of patients with PSP and HCs (Fig. 3).

**FIG 4:**
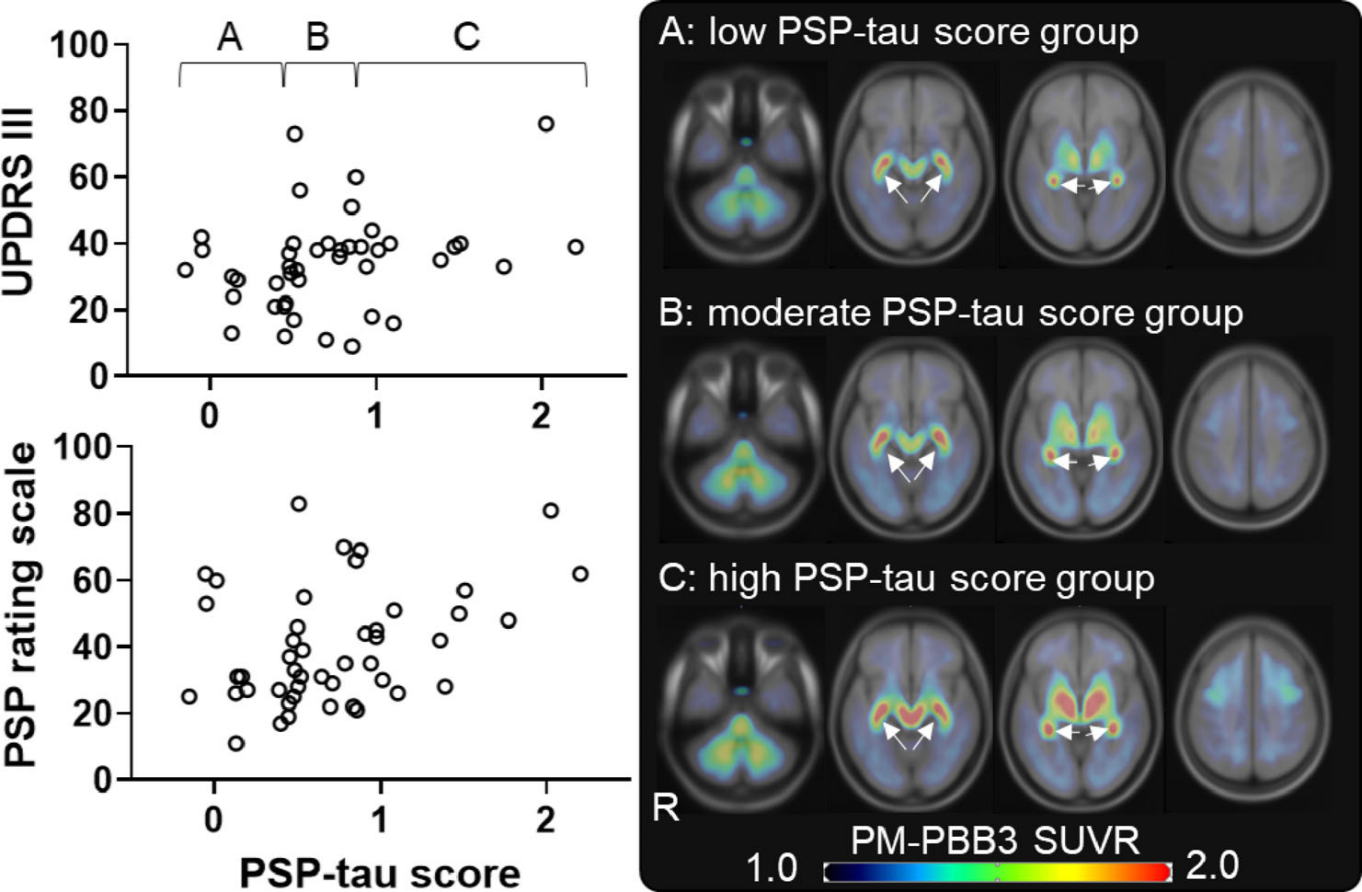
Correlation between PSP‐tau score and clinical score in PSP‐RS group. The upper left scatterplot shows the PSP‐RS group (open circles) with UPDRS III on the vertical axis and PSP‐tau score on the horizontal axis. The higher the score, the worse is UPDRS III. The lower left scatterplot shows the PSP‐RS group (open circle) with PSP rating scale on the vertical axis and PSP‐tau score on the horizontal axis. The higher the score, the worse is the PSP rating scale. The figure on the right shows the mean SUVR images of each group on the standard brain, equally divided into low PSP‐tau score group A (n = 15), moderate PSP‐tau score group B (n = 15), and high PSP‐tau score group C (n = 16) (**A**, **B**, and **C** correspond to the letters in the upper left scatterplot, respectively). The images are shown in axial sections from left to right in Montreal Neurologic Institute coordinates −36, −12, −4, and 41, respectively. Arrows indicate nonspecific accumulation in the choroid plexus. HC, healthy control; PSP, progressive supranuclear palsy; PSP‐RS, progressive supranuclear palsy–Richardson's syndrome; SUVR, standardized uptake value ratio; UPDRS III, Unified Parkinson's Disease Rating Scale Part III. [Color figure can be viewed at wileyonlinelibrary.com]

The PSP‐tau score significantly correlated with the PSP rating scale (*t*[44] = 2.38; *r*_*s*_ = 0.34; *P* = 0.02), Unified Parkinson's Disease Rating Scale [UPDRS] Part III (*t*[41] = 2.51; *r*_s_ = 0.37; *P* = 0.02), and Frontal Assessment Battery (*t*[38] = −2.27; *r*_s_ = −0.35; *P* = 0.03), which indicate the severity of PSP‐RS, and the distribution of accumulation was expanded when the scores were divided into low, moderate, and high groups (Fig. 4 and Fig. S1E). The AD‐tau score did not correlate with MMSE in the AD group, but the distribution spread as the score increased; a significant correlation was observed when participants with an MMSE ≤ 29 were included in the HC group (*t*[59] = −8.89; *r*_s_ = −0.76; *P* < 0.0001) (Fig. S3). Other clinical and neuropsychological scores were not significantly correlated with the tau scores.

## Discussion

In this study, we combined [^18^F]PM‐PBB3 tau PET with a multi‐atlas method for automatic and accurate identification of brain structures on MRI, and by using brain images of AD and PSP cases as helpful training datasets, we successfully constructed a linear model that could distinguish spatial distributions of AD‐type 3R + 4R and PSP‐type 4R tau depositions in vivo. The high discrimination performance of the machine learning approach applied to the [^18^F]PM‐PBB3 data can be interpreted as evidence of the specificity of the tracer to bind to different forms of tau fibrils (3R + 4R mixed or 4R) and its ability to localize the different pathological features of AD and PSP. The GP and medial temporal lobe were the most important regions for discriminating the PSP and AD groups, respectively. The calculated tau scores correlated with the disease characteristic severity score.

The GP, which had an important positive coefficient for discrimination, showed low tau accumulation in AD and HC, and tau pathology is considered to progress as disease severity increased.^41^ Meanwhile, tau accumulation in GP was poor in patients with PSP with a low PSP‐tau score, which could be caused by a mixture of other disease mimics, such as normal‐pressure hydrocephalus and vascular Parkinson's syndrome; it could also be that tau pathology had not yet progressed to the GP. Notably, case F in Fig. S4 showed a diffusely enlarged perivascular space (Virchow‐Robin space) in the basal ganglia (data not shown). In regions that showed negative coefficients, the amygdala showed little tau accumulation in the PSP group, as shown in Fig. 3, and little tau accumulation was reported in the genu of corpus callosum,^42^ suggesting the validity of the coefficients.

The medial temporal lobe was an important region for discrimination in AD, which was consistent with previous findings of tau PET.^43^, ^44^ There was one case in the HC group that showed faint accumulation in the medial temporal lobe despite the preservation of cognitive function, and this was thought to be a case of primary age‐related tauopathy.^45^ This case was distinguishable from AD, but the AD‐tau score was elevated because of the presence of tau accumulation (case with the highest AD‐tau score in HC in Fig. 2B = case B in Fig. S4). This result supports a prior report that medial temporal lobe tau without PET‐detectable cortical Aβ may reflect a very early stage of the AD pathological continuum.^46^ These results suggest that this tau scoring system can objectively capture the accumulation of [^18^F]PM‐PBB3 in pathologically important areas, even in the presence of accumulation that may indicate nonspecific binding to the choroid plexus, which is close to the medial temporal lobe.

Tau PET accumulation and MMSE in AD often do not correlate or require several efforts to find a correlation,^47^, ^48^, ^49^, ^50^, ^51^ but when the continuous amount of scores using the AD‐tau scoring system was examined, including HCs with even a single‐point decrease (MMSE score ≤ 29), a correlation was found even in MMSE, and the distribution of [^18^F]PM‐PBB3 accumulation expanded as the score increased (Fig. S3). Furthermore, the correlation between MMSE and AD‐tau scores for HCs with an MMSE score ≤29 and MCI alone (*t*[35] = −6.62; *r*_s_ − 0.75; *P* < 0.0001) (Table S3) and the lack of correlation in HCs with an MMSE score ≤29 (*t*[23] = 0.35; *r*_s_ − 0.19; *P* = 0.35) or all HCs (*t*[48] = −1.11; *r*_s_ − 0.15; *P* = 0.27) suggest that AD‐tau scores can be used to measure disease severity from HC to MCI and is also important for discriminating between the AD continuum and HCs. In the AD group, the coefficient of the cerebellar region was negative, suggesting that high tau accumulation in the cerebellum does not support a diagnosis of AD.

In cases with low PSP‐tau scores in PSP‐RS, the PSP rating scale was also low. Future longitudinal evaluation and the development of early tau scores for these groups are considered important. Notably, one HC case with a PSP‐tau score above the cutoff had a PSP rating scale of five points (eye, two points; limb, three points) and Frontal Assessment Battery of 14 points, suggesting the possibility of a prodromal and/or subclinical state (Fig. S4, case A). This finding further highlights the importance of longitudinal research and an early tau scoring system. In addition, the PSP‐tau score was lower in cases showing a strong accumulation pattern of left–right differences (Fig. S4, cases F, H), suggesting the possibility of calculating the corticobasal degeneration (CBD)‐tau score by adding information regarding left–right differences, such as asymmetry index, to machine learning if sufficient data on CBD cases are obtained. Therefore, there is a possibility that the CBD‐tau score could be calculated in the future.^52^ Consideration of mixed pathology is also necessary because the PSP‐tau score may be underestimated, eg, cases where AD pathology is accompanied by PSP pathology. Although this study focused on discrimination, we were able to show the correlation with severity. In the future, we aim to develop a severity‐specific scoring system for populations with tau scores higher than the cutoff, or an early diagnosis‐specific diagnostic system, by collecting data from individuals with lower tau scores and adding other features.

In conclusion, we generated and validated an objective imaging‐based neuropathological score that quantitatively indicated the presence of either 3R + 4R or 4R tauopathies in each individual by applying the Elastic Net model on [^18^F]PM‐PBB3‐PET data from the PSP and AD continuum. These indices can be used as pathology indicators for PSP and AD, which may be useful for robust recruitment and treatment evaluation in clinical trials of disease‐modifying drugs because they also provide information regarding disease severity.

## Author Roles

(1) Research Project: A. Conception and Design; B. Acquisition of Data; C. Analysis and Interpretation of Data; (2) Manuscript: A. Writing of the First Draft; B. Review and Critique; (3) Other: A. Statistical Design; B. Processing and Analysis of Pathological Specimens; C. Methods for Data Analysis; D. Synthesizing of Radioligands.

H.E.: 1A; 1B; 1C; 2A; 3A

K. Tagai: 1A; 1B; 2B

M. Ono: 2B; 3B

Y.I.: 2B; 3A; 3C

A.O.: 3C

K.M.: 1B; 2B

N.K.: 1B; 2B

K.H.: 1B; 2B

Y.S.: 1B; 2B

M. Oya: 1B; 2B

H.M.: 1B; 2B

S.K.: 1B; 2B

C.S.: 1C; 2B

H. Shimizu: 3B

A.K.: 3B

K. Takahata: 1B; 2B

H. Shinotoh: 2B

H. Shimada: 1B; 2B

T.T.: 2B

K.K.: 2B; 3D

M.‐R.Z.: 2B; 3D

K.O.: 2B; 3C

S.M.: 2B; 3C

Y.T.: 1A; 1C; 2B; 3C

M.H.: 1A; 1C; 2B; 3C

## Financial Disclosures for the Previous 12 Months

H.E. is employed by QST, is supported by JSPS KAKENHI, and is a consultant on image analysis for APRINOIA Therapeutics. K. Tagai, Y.I., K.M., N.K., and K.K. are employed by QST and are supported by JSPS KAKENHI. M. Ono and Y.T. are employed by QST and supported by JSPS KAKENHI and AMED. A.O. and C.S. are employed by QST. K.H. is supported by JPSP KAKENHI. Y.S., M. Oya, and S.K. report no disclosures. H.M. is supported by the Tokyo Dental College Research Branding project. H. Shimizu and A.K. are employed by Niigata University and are supported by JSPS KAKENHI. K. Takahata is employed by QST and is supported by JSPS KAKENHI and MHLF JPMH. H. Shinotoh is employed by Neurology Clinic Chiba. H. Shimada is employed by Niigata University, is supported by JSPS KAKENHI, and holds patents (JP 5422782/EP 12 884742.3/CA2894994/HK1208672/ZL201710407246.4). T.T is employed by QST and is supported by AMED. M.R.Z. is employed by QST, is supported by JSPS KAKENHI and AMED, and holds patents (JP 5422782/EP 12 884 742.3/CA2894994/HK1208672/ZL201710407246.4). K.O. is employed by Johns Hopkins University and is a consultant of AnatomyWorks and M Corporation. S.M. is employed by Johns Hopkins University, is a co‐founder of AnatomyWorks and M Corporation, and is CEO of AnatomyWorks. M.H. is employed by QST, is supported by JSPS KAKENHI, AMED, and JST CREST, and holds patents (JP 5422782/EP 12 884 742.3/CA2894994/HK1208672/ZL201710407246.4).

## Supporting information


**Appendix S1** Supporting Information.

**Table S1.** M‐Vision brain abbreviations.
**Table S2.** Demographics of the cases used for the histological examination.
**Table S3.** Demographics with a breakdown of the AD continuum.

**Fig. S1.** Schematic of the analysis flow.

**Fig. S2.** Weighting coefficients of ROIs with high contribution to discrimination between AD group and PSP‐RS + HC group.

**Fig. S3.** Representative examples for each PSP‐tau score.

**Fig. S4.** Correlation between AD‐tau score and MMSE in HC group with slightly decreased MMSE and AD group.

## Data Availability

The data that support the findings of this study are available from the corresponding author upon reasonable request.
