# Supplementary material for: A Machine Learning–Based Approach to Discrimination of Tauopathies Using [ 18F]PM‐PBB3 PET Images
Source: Mov Disord. 2022 Aug 28;37(11):2236–46. doi: 10.1002/mds.29173 (PMC9805085; doi:10.1002/mds.29173)
Supplement: Supplementary file 1 — Appendix S1 Supporting Information. [file MDS-37-2236-s003.docx]

**Supplementary materials**

Supplementary material associated with this article can be found in the online version.

**eMethods**

***Neuropsychological tests***

The participants underwent neurological examinations, including unified Parkinson’s disease rating scale part III (UPDRS III) and progressive supranuclear palsy (PSP) rating scale for assessing motor symptoms and psychological evaluation, including Mini-Mental State Examination (MMSE), Clinical Dementia Rating Scale (CDR), CDR sum of Boxes (CDR-SOB), and Montreal Cognitive Assessment (MoCA) for assessing cognitive and functional impairment, geriatric depression scale (GDS) for assessing depression, apathy scale for assessing motivational less, and frontal assessment battery (FAB) for assessing frontal dysfunction. The above-mentioned test batteries are presented in Table 1.

***Radioligand synthesis***

Radiosynthesis of [^18^F]PM-PBB3 and [^11^C]PiB was performed as described elsewhere^1-3^. Briefly, [^18^F]-fluoride reacted with tosylate precursor of PM-PBB3 in the presence of dimethyl sulfoxide, K_2_CO_3_, and K222. The final formulated product of [^18^F]PM-PBB3 was radiochemically pure (≥ 95%) due to the use of analytic high-performance liquid chromatography (Waters Atlantis prep T3 column, 4.6 × 150 mm; CH_3_CN/50 mM AcONH_4_ = 4/6, 1 ml/min). Specific activities of [^18^F]PM-PBB3 and [^11^C]PiB at the time of injection were 73.4–546.5 and 27.0–340.8 GBq/μmol, respectively.

***Histological examinations***

The pathological sections used for histological examination were collected from other studies and their demographics are shown in Supplemental Table 2. For fluorescence labeling, deparaffinized sections were incubated in 50% ethanol containing 30 μM of non-radiolabeled PM-PBB3 at room temperature and rinsed with 50% ethanol for 5 min, dipped into distilled water twice for 3 min, and mounted on non-fluorescent mounting media (VECTASHIELD, Vector Laboratories). Fluorescence images were captured using a DM4000 microscope (Leica) equipped with a custom filter cube for PBB3 (excitation band-pass at 414/46 nm and suppression low-pass with 458 nm cutoff)^4^. Following microscopy, sections were autoclaved for antigen retrieval and immunostained with AT8. Immunolabeling was then examined using DM4000. Finally, the tested samples were used for (Gallyas-Braak) GB staining with Nuclear Fast Red (Sigma-Aldrich) counter-staining after pretreatment with 0.25% KMnO4 followed by 2% oxalic acid.

***Data preprocessing***

Data preprocessing was performed using PMOD 4.2 (PMOD Technologies LLC, Switzerland), Statistical Parametric Mapping software (SPM12, Wellcome Department of Cognitive Neurology), RStudio (Version 1.3.1056, RStudio, PBC), and M-Vision brain (M Corporation, Japan). To obtain the T1WI segmentation map, M-Vision brain was performed to down sample the matrix of T1WI from 512 × 512 × 176 to 256 × 256 × 176. SUVR images were generated from averaged PET images with motion correction at the following intervals: 50–70 min ([^11^C]PiB) and 90–110 min ([^18^F]PM-PBB3) postinjection, respectively. Cerebellar gray matter was used as reference region for [^11^C]PiB PET. We used a new algorithm in-house script implemented MATLAB (The Mathworks, Natick, MA, USA), which automatically extracted the gray matter (GM) reference region by the signal histogram, as a reference region for [^18^F]PM-PBB3^5^. The acquired down-sampled T1WI and segmentation map were rigidly coregistered to individual [^18^F]PM-PBB3 SUVR images. Regarding the average image creation of [^18^F]PM-PBB3 SUVR according to the severity of the disease, each image (T1WI and SUVR) was spatially normalized to MNI (Montreal Neurologic Institute) space (East Asian brain T1WI from International Consortium for Brain Mapping) using Diffeomorphic Anatomical Registration Through Exponentiated Lie Algebra (DARTEL) algorithm.

***Elastic Net***

Machine learning was performed using the Python code described in “Machine Learning with Spark and Python: Essential Techniques for Predictive Analytics” by Michael Bowles^6^. PSP-RS versus HC+AD and AD versus HC+PSP-RS were solved as a linear binary classification problem to determine zero or one, respectively.

In a regression where the target value is expected to be a linear combination of features, **ŷ** should be the predicted value


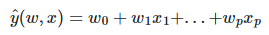
(Eq. 1)

The objective function to be minimized in the Elastic Net is as follows:


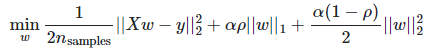
(Eq. 2)

We denote the vector **ω = (ω_1_, … , ω_p_)** as a coefficient, **ω_0_** as intercept, and **ρ** as L1_ratio. Elastic Net is a linear regression model trained with both L1-norm regularization (second term of the Eq. 2) and L2-norm regularization (third term of the Eq. 2) of the coefficients; L1_ratio=1 corresponds to Least Absolute Shrinkage and Selection Operator (LASSO). The combination of L1 and L2 convexity is controlled by the L1_ratio parameter. LASSO is more likely to randomly select one of the features, whereas Elastic Net is more likely to select both. We tried values from 0.01 to 1.0 to determine the optimal L1_ratio, and we chose the one with the largest L1_ratio and the largest AUC for each of the 10 sets of training data^7^ (Supplemental Figure 1a).


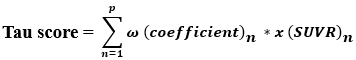
The equation for the linearly combined tau score (supplemental Figure 1d, 1e) is

(Eq.3)

**Supplementary Results**

***A preliminary assay with corrections for partial volume effects***

We did not perform partial volume correction (PVC) in this study despite the regional atrophy of the AD and PSP brains to varying extent, as an optimal method for PVC has not yet been established. Specifically, how the PVC and subsequently our Elastic Net model are affected by nonspecific accumulations of [^18^F]PM-PBB3 in the choroid plexus remains to be clarified. Unless a consensus procedure is established, PVC may not be incorporated into the automated image processing system that will be applicable to multicenter studies. To examine the influences of PVC on the results, we compared the data uncorrected for atrophy and corrected for partial volume effects using the geometric transfer matrix method^8^ as a preliminary assay. In this study, the performance of these two methods for the differentiation between the diagnostic groups was nearly comparable, with the inclusion of PVC yielding AUCs of 0.985 for the separation of PSP-RS and AD + HC groups and 1.000 for the separation of AD and the PSP-RS + HC groups. Therefore, we postulate that our Elastic Net model is not susceptible to the effects of partial volume.

| Models | AUC | 95% CI | P value | Cutoff | Accuracy | Sensitivity | Specificity |
| --- | --- | --- | --- | --- | --- | --- | --- |
| PSP-RS  versus  AD+HC | 0.982 | 0.9641 to 0.9999 | <0.0001 | 0.1143 | 0.955 | 0.913 | 0.977 |
| PSP-RS  versus  AD+HC  **with PVC** | 0.985 | 0.9682  to  1.000 | <0.0001 | 0.0483 | 0.970 | 0.913 | 1.000 |
| AD  versus  PSP-RS+HC | 1.000 | 1.000  to  1.000 | <0.0001 | 0.3431 | 1.000 | 1.000 | 1.000 |
| AD  versus  PSP-RS+HC  **with PVC** | 1.000 | 1.000  to  1.000 | <0.0001 | 0.1664 | 1.000 | 1.000 | 1.000 |

CI: confidence interval

***Performance of quantification with extracted GM reference voxels in comparison with conventional cerebellar cortical reference and with inclusion and exclusion of the HC group***

In this study, GM voxels extracted based on a frequency histogram were used as reference tissue for the calculation of SUVRs. When SUVRs were determined using the conventional cerebellar GM reference, the machine learning algorithm-based scoring allowed discrimination between the PSP-RS and AD + HC groups with an AUC of 0.978 and the AD and PSP-RS + HC groups with an AUC of 1.000. Hence, the performance of the method with the cerebellar GM reference in the differentiation of the PSP-RS group appears inferior to that of the methods with extracted GM reference voxels, possibly because of the spillover of the cerebellar white matter radioactivity into the cerebellar GM.

We have also compared the performances of AD-tau and PSP-tau scores for the separation of AD and PSP-RS cases by excluding HCs. As displayed in the table below, these two scores yielded similar AUC, accuracy, sensitivity, and specificity.

| Models | Ref. | AUC | 95%  CI | P value | Cutoff | Accuracy | Sensitivity | Specificity |
| --- | --- | --- | --- | --- | --- | --- | --- | --- |
| PSP-RS  versus  AD+HC  by PSP-tau score | HG | 0.982 | 0.96  to  1.00 | <0.0001 | 0.11 | 0.955 | 0.913 | 0.977 |
| PSP-RS  versus  AD+HC  by PSP-tau  score | **CB** | 0.978 | 0.96  to  1.00 | <0.0001 | 0.25 | 0.940 | 0.848 | 0.989 |
| PSP-RS  versus  AD  **without HC**  by PSP-tau  score | HG | 0.997 | 0.99  to  1.00 | <0.0001 | -0.19 | 0.976 | 1.000 | 0.946 |
| AD  versus  PSP-RS  **without HC**  by AD-tau  score | HG | 1.000 | 1.00  to  1.00 | <0.0001 | 0.17 | 1.000 | 1.000 | 1.000 |
| AD  versus  PS-PRS+HC  by AD-tau score | HG | 1.000 | 1.00 to  1.00 | <0.0001 | 0.34 | 1.000 | 1.000 | 1.000 |
| AD  versus  PSP-RS+HC  by AD-tau  score | **CB** | 1.000 | 1.00 to  1.00 | <0.0001 | 0.25 | 1.000 | 1.000 | 1.000 |

HG: Histogram (Gray matter), CB: Cerebellar gray matter, Ref.: Reference

**Supplementary Discussion**

***Use of subthalamic nucleus (STN) radioligand retention as a discrimination index***

In this study, we used the M-Vision brain to segmentalize the brain. However, this does not include the STN, a major region involved in the PSP tau pathology. The signal intensity of the STN may not be readily distinguishable from other regions in T1WI images due to insufficient MRI contrast and the small volume of this anatomical structure. In this study, the combined incorporation of other regions in the machine learning-based discrimination resulted in a better performance than the use of SUVR in the STN alone as an index^2, 5^.

***Applicability of the current technology to other Parkinsonian syndromes***

Corticobasal syndrome (CBS) can be classified into putative AD and non-AD diseases according to the amyloid PET findings, whereas more detailed subcategories of CBS may be provided by a radioligand for 3R+4R and 4R tau fibrils. Our first-generation probe, [^11^C]PBB3, helps identify AD, corticobasal degeneration (CBD), PSP, and non-tau-disorders in individuals with CBS^9^. Thus, clear discriminations of these illnesses based on disease-specific tau scores will be achieved by applying high-contrast tau [^18^F]PM-PBB3-PET images to the current Elastic Net model, which could be expanded to the *in vivo* pathology-based differentiation of Parkinson’s disease and atypical Parkinsonian syndromes, including CBD, PSP, and multiple system atrophy that predominated in parkinsonism (MSA-P). The regionally distinct [^18^F]PM-PBB3 retentions in PSP and MSA-P cases were also documented in a recent study^10^, although the binding components for the radioligand in MSA-P require further evaluation.

***Analysis of correlations between tau scores and clinical and psychological data corrected for age and sex***

We did not find any correlations between AD-tau and MMSE scores in the AD continuum subjects without controlling demographic parameters. However, we found a correlation between these two measures in the AD continuum cases when we linearly corrected MMSE scores for the age and sex as we applied to a preprocessing step in the calculation of tau scores [t(34) = −2.06, *r_s_* −0.33, *p* = 0.047]. The MoCA was excluded from the correlation analysis due to the limited number of participants who took the assessment. For a similar region, the correlation of PSP-tau scores with the Clinical Dementia Rating Scale (CDR) and CDR sum of boxes in the PSP group was not examined. In the AD group, the correlation between AD-tau and motor symptom scores was not examined. We also found that AD-tau scores were not correlated with other neuropsychological measures corrected for age and sex in the AD continuum group.

**Supplemental References**

1. Maruyama M, Shimada H, Suhara T, et al. Imaging of tau pathology in a tauopathy mouse model and in Alzheimer patients compared to normal controls. Neuron 2013;79(6):1094-1108.

2. Tagai K, Ono M, Kubota M, et al. High-Contrast In Vivo Imaging of Tau Pathologies in Alzheimer's and Non-Alzheimer's Disease Tauopathies. Neuron 2021;109(1):42-58 e48.

3. Kimura Y, Ichise M, Ito H, et al. PET Quantification of Tau Pathology in Human Brain with 11C-PBB3. Journal of nuclear medicine : official publication, Society of Nuclear Medicine 2015;56(9):1359-1365.

4. Ono M, Sahara N, Kumata K, et al. Distinct binding of PET ligands PBB3 and AV-1451 to tau fibril strains in neurodegenerative tauopathies. Brain : a journal of neurology 2017;140(3):764-780.

5. Tagai K, Ikoma Y, Endo H, et al. An optimized reference tissue method for quantification of tau protein depositions in diverse neurodegenerative disorders by PET with ^18^F-PM-PBB3 (^18^F-APN-1607). MedRxiv 2022.

6. Bowles M. Machine Learning with Spark and Python, 2nd Edition. 2019.

7. Pedregosa F, Varoquaux G, Gramfort A, Michel V, Thirion B. Scikit-learn: Machine Learning in Python. Journal of Machine Learning Research 2011;12:2825-2830.

8. Rousset OG, Ma Y, Evans AC. Correction for partial volume effects in PET: principle and validation. Journal of nuclear medicine : official publication, Society of Nuclear Medicine 1998;39(5):904-911.

9. Nakano Y, Shimada H, Shinotoh H, et al. PET-based classification of corticobasal syndrome. Parkinsonism & related disorders 2022;98:92-98.

10. Li L, Liu FT, Li M, et al. Clinical Utility of (18) F-APN-1607 Tau PET Imaging in Patients with Progressive Supranuclear Palsy. Movement disorders : official journal of the Movement Disorder Society 2021;36(10):2314-2323.
