# Supplementary figures and images for: A Machine Learning–Based Approach to Discrimination of Tauopathies Using [ 18F]PM‐PBB3 PET Images
Source: Mov Disord. 2022 Aug 28;37(11):2236–46. doi: 10.1002/mds.29173 (PMC9805085; doi:10.1002/mds.29173)

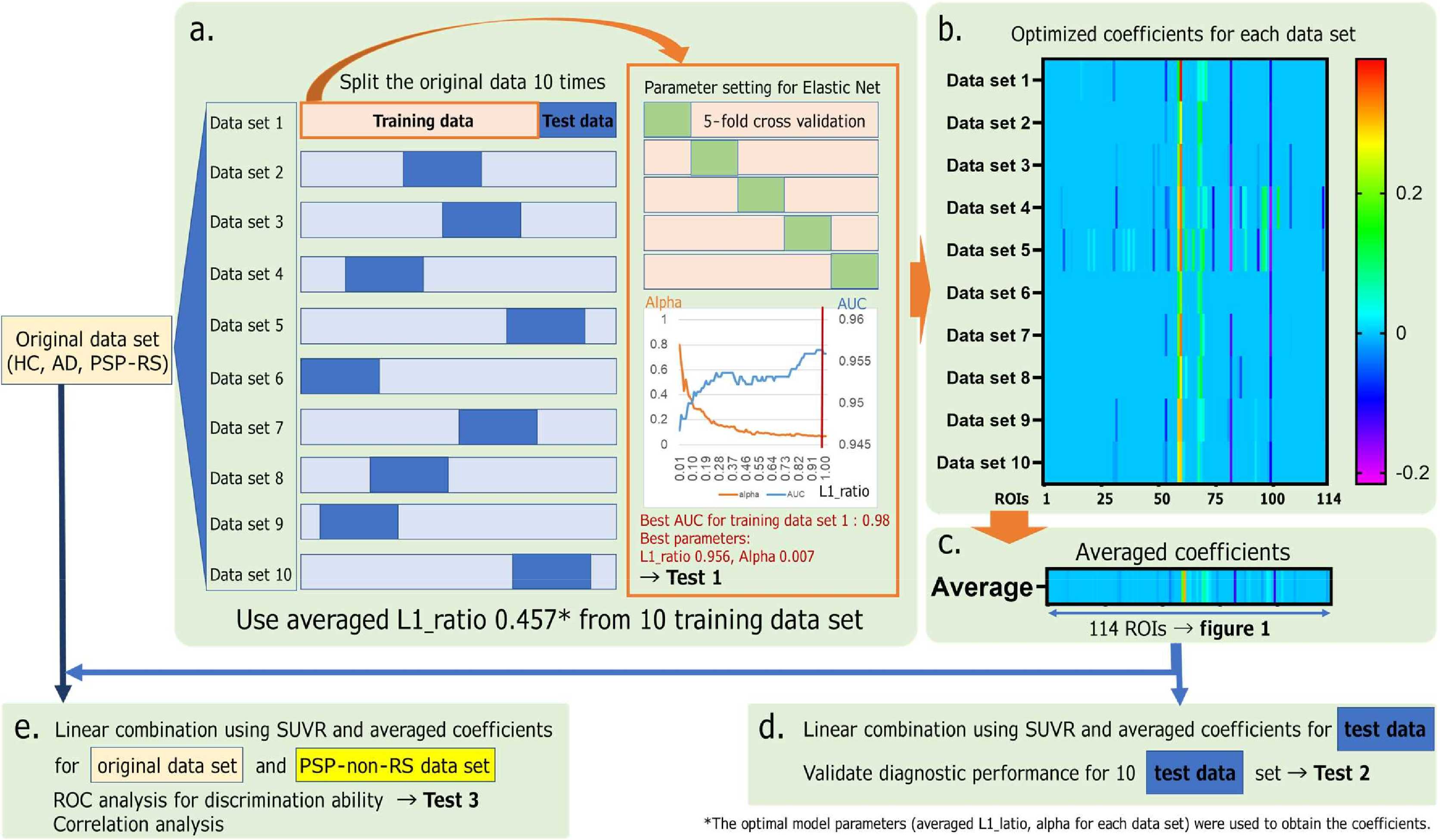

Supplement: Supplementary file 3 — Fig. S1. Schematic of the analysis flow. [file MDS-37-2236-s001.tif]

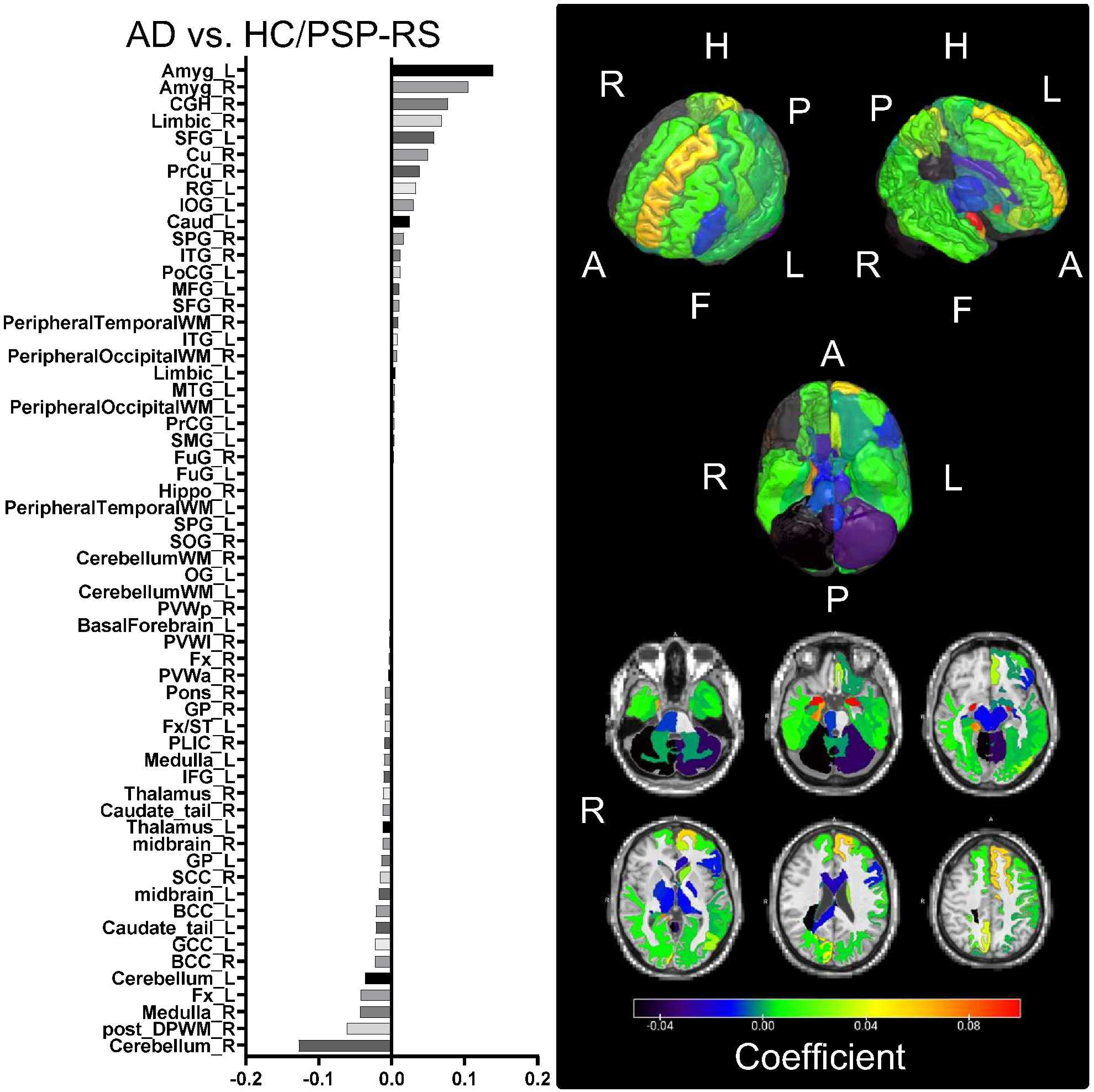

Supplement: Supplementary file 4 — Fig. S2. Weighting coefficients of ROIs with high contribution to discrimination between AD group and PSP‐RS + HC group. [file MDS-37-2236-s002.tif]

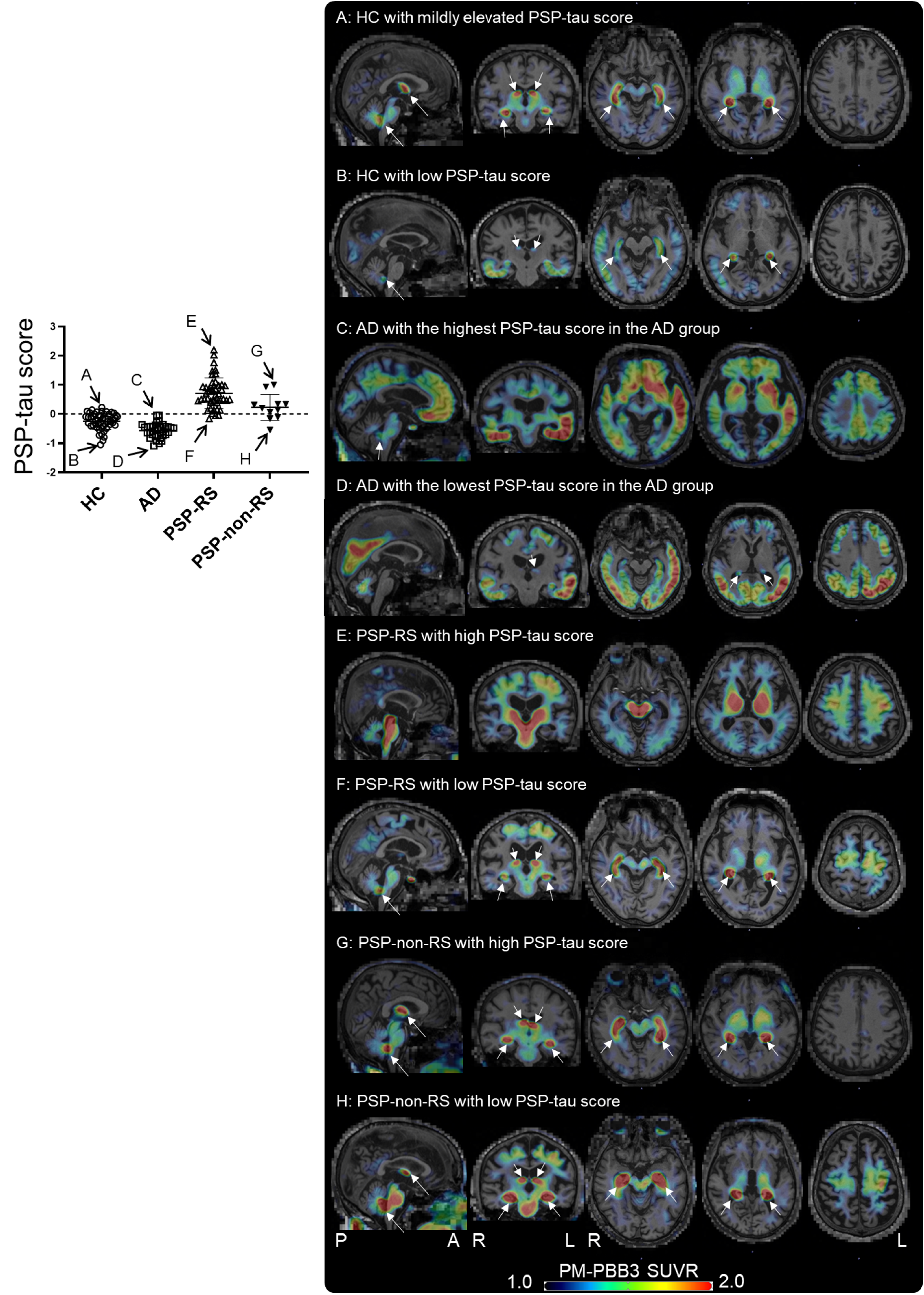

Supplement: Supplementary file 5 — Fig. S3. Representative examples for each PSP‐tau score. [file MDS-37-2236-s005.tif]

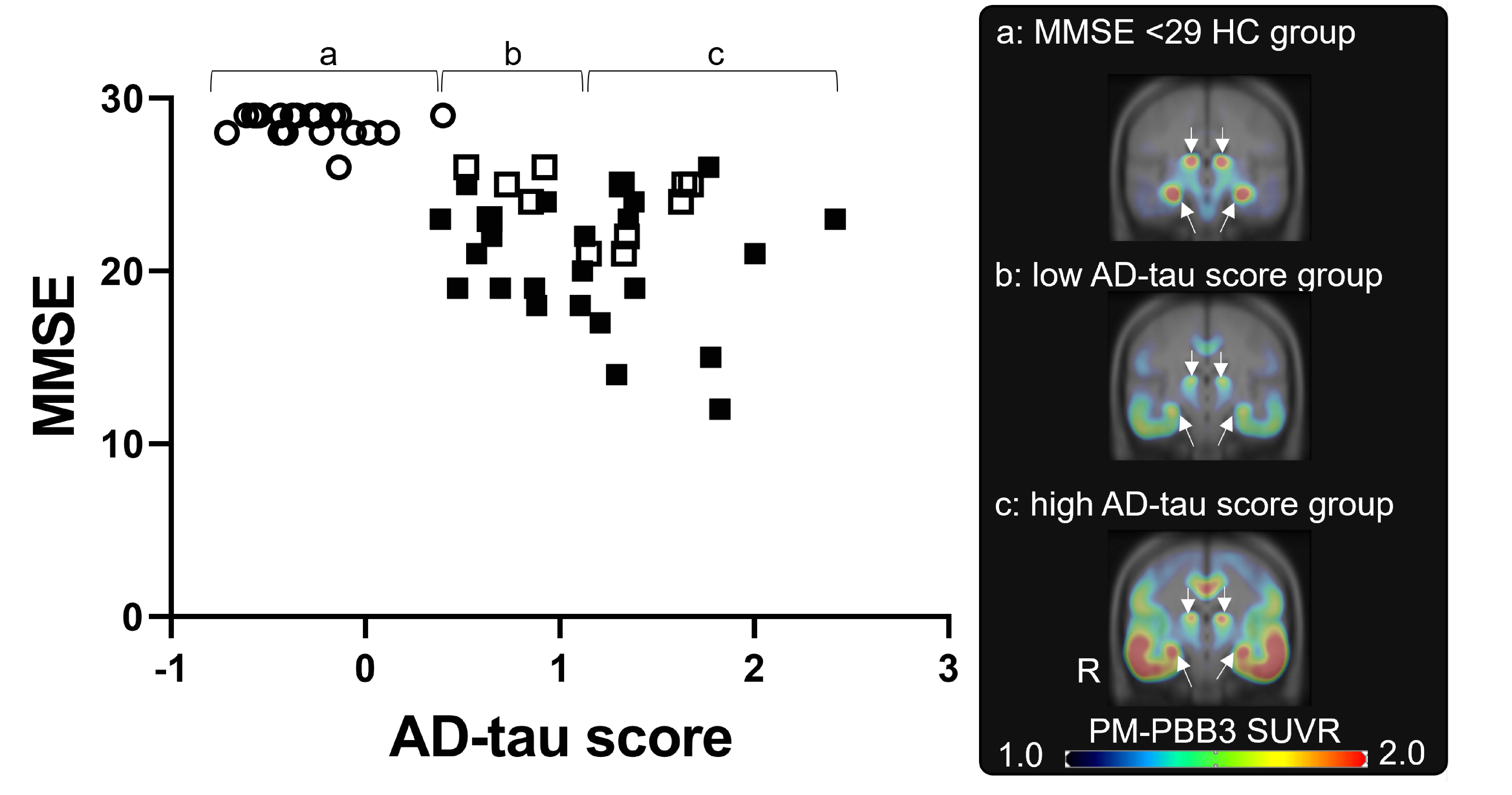

Supplement: Supplementary file 6 — Fig. S4. Correlation between AD‐tau score and MMSE in HC group with slightly decreased MMSE and AD group. [file MDS-37-2236-s006.tif]
